# Omega-3 Fatty Acids and Fecundation, Pregnancy and Breastfeeding

**DOI:** 10.1055/s-0040-1708090

**Published:** 2020-03

**Authors:** Carlos Alberto Politano, Jorge López-Berroa

**Affiliations:** 1 Departament of Gynecology and Obstetrics, Sociedade de Medicina e Cirurgia de Campinas, São Paulo, Brazil; 2 Latam Exeltis, Ciudad de Panamá, Panama

**Keywords:** omega-3, eicosapentaenoic acid, docosahexaenoic acid, pregnancy, breastfeeding, ômega 3, ácido eicosapentaenoico, ácido docosa-hexanoico, gravidez, amamentação

## Abstract

Long-chain omega-3 (n-3) polyunsaturated fatty acids (PUFAs), such as the eicosapentaenoic and docosahexaenoic acids, have been linked to human health in all stages of life, from fetal development to aging. These PUFAs act as precursors for various metabolites involved in the prevention of certain diseases. The recognizable effects of these supplements prior to pregnancy (oocyte maturation), during pregnancy (improvement in the risk of premature delivery, among others) and in the offspring (in terms of cognitive function and the approach to neurodevelopmental disorders) are described in the present narrative review. We concluded that the diffusion of these supplements may improve the prognosis of these patients in a simple, effective way, and with high safety rates.

## Resumo

Os ácidos graxos poli-insaturados (AGPIs) ômega-3 (n-3) de cadeia longa, como os ácidos eicosapentaenoico e docosa-hexaenoico, têm sido associados à saúde humana em todas as etapas da vida, do desenvolvimento fetal ao envelhecimento. Esses AGPIs atuam como precursores de vários metabólitos envolvidos na prevenção de algumas doenças. Os efeitos reconhecíveis desses suplementos antes da gravidez (maturação dos oócitos), durante a gravidez (melhora do risco de parto prematuro, entre outros) e nos descendentes (em termos de função cognitiva e abordagem dos distúrbios do neurodesenvolvimento) são apresentados nesta revisão narrativa. Concluiu-se que a difusão desses suplementos pode melhorar o prognóstico desses pacientes de maneira simples, eficaz, e com altas taxas de segurança.

## Introduction

Long-chain omega-3 (n-3) polyunsaturated fatty acids (PUFAs), such as the eicosapentaenoic (EPA) and docosahexaenoic acids (DHA) have been linked to human health in all stages of life, from fetal development to physiological aging.^1^ The DHA is a relevant component of all biological membranes, and both of these PUFAs are precursors for various metabolites involved in the prevention of several diseases.^1^

However, in many cases, the contribution of a diet rich in fish is not enough to ensure an adequate intake of these PUFAs.^1^ Human tissues express enzymes that can metabolize the alpha-linolenic acid (ALA) for the synthesis of DHA and EPA, but in a reduced proportion.^2^ As a consequence, in many occasions supplementation may be necessary.

The objective of the present review is to describe the potential benefits of DHA and EPA supplementation in the context of reproductive medicine.

## Before Conception

Oocyte quality is identified as one of the most relevant factors in relation to female fertility, not only for the fertilization process, but also to ensure embryonic implantation and development.^3^ Among the factors related to oocyte quality, maternal diet is highlighted, since obesity and diabetes can alter mitochondrial function and chromosome alignment at the gamete level.^4^ Accordingly, the concentration of fatty acids in the ovarian follicular microenvironment is associated with oocyte quality and embryonic development during *in vitro* fertilization procedures.^4^

**Fig. 1:**
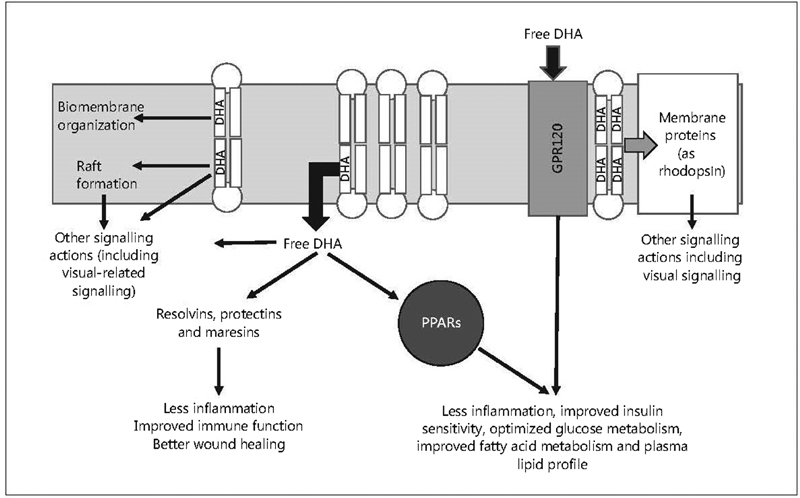
Molecular mechanisms and signaling of n-3 P. Source: Adapted and modified from Calder.^6^

The follicular levels of n-3 PUFAs are correlated with plasma lipids, which are modified according to diet and body weight.^4^ It is hypothesized that n-3 PUFAs can regulate oocyte maturation by mechanisms mediated by functional changes in granulosa cells linked to peroxisome-proliferator-activated receptors (PPARs) and modifications in the biosynthesis of follicular prostaglandins.^4^ According to experimental data, PUFAs are potent PPAR ligands and also increase the expression of the steroidogenic acute regulatory protein (STAR). Thus, a correlation between the circulating levels of PUFAs and the concentration of sex hormones is verified in humans.^5^ On the other hand, sex steroids can modulate the synthesis of PUFAs by modifying the expression of desaturases (Figure 1).^6^

These data from preclinical and experimental models were corroborated by clinical studies. In a prospective trial^7^ involving 100 women under assisted reproduction treatment (total: 136 cycles), the levels of circulating n-3 PUFAs were associated with a higher probability of clinical pregnancy and a higher number of live births, and PUFA supplementation was also related with a higher probability of births.^7^

On the other hand, PUFAs have been linked to an optimization of spermatogenesis. In animal models, it was shown that PUFA supplementation stimulates the development of seminiferous tubules and increases the density of Sertoli cells.^8^ An improvement in the quality and quantity of sperm cells was also reported.^9^

In humans, a high concentration of DHA in the spermatozoon head is recognized; the dietary content of fatty acids appears to modify the profile of these molecules in male gametes.^10^ However, the effects of n-3 PUFA supplementation are described after no less than 4 weeks of administration; in addition, these effects seem dose-dependent.^10^

## Use during Pregnancy

It has been shown that the conversion rate of ALA into EPA and DHA in men reaches 8% and less than 1% respectively, while in women the corresponding rates are 21% and 9% respectively.^6^ This difference is attributed to the higher requirements of n-3 PUFAs during pregnancy and breastfeeding.^6^ According to the joint recommendations of the World Association of Perinatal Medicine, the Early Nutrition Academy and the Child Health Foundation, maternal DHA consumption during pregnancy should not be lower than 200 mg/day. Nutritional supplements are one of the suggested sources to achieve this goal.^11^

Even though a diet rich in fish is also a source of DHA/EPA, the potential content of contaminants, such as mercury, is a matter of concern.^12^ Thus, supplements with adequate processing quality to avoid impurities and contaminants may play a prominent role.^12^ The administration of between 200 mg and 300 mg/day of DHA to this population has been endorsed by the National Institutes of Health and the International Society for the Study of Fatty Acids and Lipids, among other specialized societies.^12^

The maximum growth of the human brain occurs from the beginning of the third trimester of intrauterine life until 18 months after birth;^6^ during this period, the concentration of DHA increases significantly from 900 µg/g to 3,000 µg/g.^6^ Therefore, the need for an adequate n-3 PUFA contribution during this period is highlighted, in order to ensure a normal growth and visual and neurological developments.^6^ Although there is a correlation between DHA levels in maternal and fetal blood, this PUFA is concentrated in the circulation and fetal tissues due to a process known as biomagnification, which seems mediated by the placenta.^6^

Another benefit associated with the PUFA n-3 supplement is the prevention of preterm births (PBs). This is highly important, considering that PB accounts for more than 85% of all perinatal mortality and complications,^13^ in addition to accounting for a greater requirement of intensive care unit resources and a higher risk of early comorbidities, with potential consequences throughout life.^13^ Nevertheless, there are no primary prevention strategies frequently used in the daily practice. In a systematic review with meta-analysis^14^ in which the risk of PB and extreme PB (less than 34 weeks of gestation) was evaluated among 5,980 women, the authors verified that the n-3 PUFA supplementation during pregnancy significantly reduced the risk of both criteria. Likewise, they confirmed that these supplements were linked to an increase in birth weight of around 122 g when the participants were compared with those who were not submitted to the supplementation.^14^

Taking into account its ease of administration and the achieved results, n-3 PUFA supplementation could play a role as a preventive strategy for PB at a population level, independently of the previous risk and of the dose administered.^14^

**Fig. 2:**
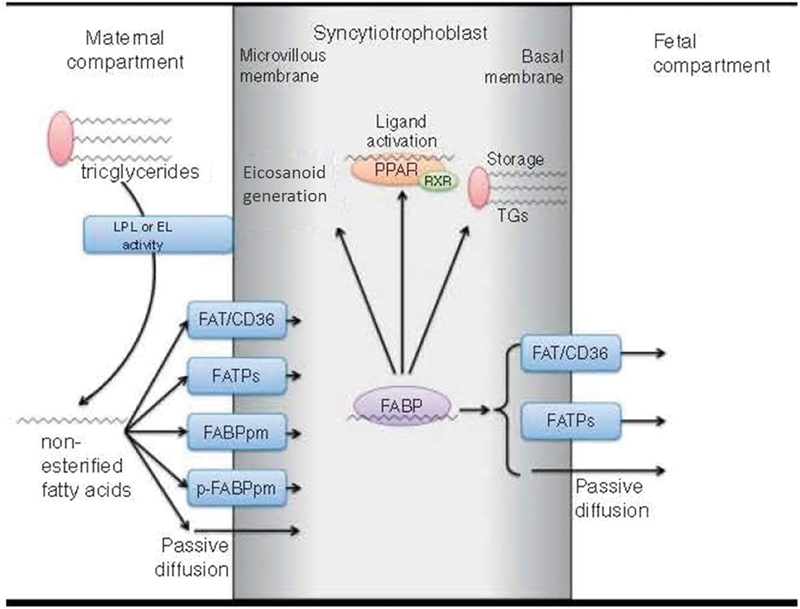
Mechanisms of transplacental omega-3 (n-3) polyunsaturated fatty acid (PUFA) transport. Source: adapted and modified from Jones et al.^17^ Abbreviations: EL, epithelial lipase; FABP, fatty-acid-binding protein; FABPpm, plasma membrane FABP; FAT/CD36, fatty acid translocase; FATPs, fatty acid transport proteins; LPL, lipoprotein lipase; p-FABPpm, placental plasma membrane FABP; PPAR, peroxisome-proliferator-activated receptor; RXR, retinoic-acid-X receptor; TGs, triglycerides.

Moreover, it has been reported that n-3 PUFAs have beneficial effects in women with gestational diabetes, which also extend to the fetus.^15^ In cases of gestational diabetes and preeclampsia, transplacental transmission of n-3 PUFAs is reduced, as demonstrated by lower levels of DHA detected in umbilical cord blood, compared to pregnancies without these alterations.^16^ The importance of early DHA supplementation in women with gestational diabetes is evident, since its late administration would not improve fetal circulation levels, due to the already established alterations in the placental transfer (Figure 2).^17^

Therefore, the appropriate approach to gestational diabetes, in association with fatty-acid supplementation, may improve the metabolic and immune functions, and decrease the risk of PB and the likelihood of developing preeclampsia and neonatal and long-term infant complications.^15^

## Administration during Breastfeeding: Cognitive Effects

Given the importance of DHA for the development of the neonate and infant brain, the importance of this PUFA during breastfeeding is highlighted. The suggested doses are similar to those proposed during pregnancy.^11^ ^12^ The levels of DHA in breast milk may be increased by supplementation,^6^ and they seem to correlate with cognitive function and language development in infants, as well as with general psychosocial health.^18^ The daily intake of DHA by breastfed infants is estimated between 13 mg to 26 mg/day, which is lower than the intrauterine supply (45–50 mg/kg/day).

The clinical evidence available supports that n-3 PUFA supplementation may optimize the neuronal development of term infants.^18^ PBs have been linked with deficiencies in the integrity of the connectivity of the myelin and cortical circuitry, among other complications.^18^ These neurodevelopmental alterations increase the risk of autism spectrum disorders (ASDs) and attention deficit and hyperactivity disorder (ADHD).^18^ Low DHA levels may at least partially explain these outcomes:^18^ in a randomized, placebo-controlled study involving 141 infants with birth weight lower than 1,500 g, the addition of 32 mg of DHA and 31 mg of arachidonic acid per 100 ml of human milk from the first week of life, for an average of 9 weeks, was associated with an improvement in recognition memory and ability to solve problems at 6 months.^19^ It is hypothesized that conventional DHA ingestion may be insufficient in PB children, since these patients are more sensitive to the effects of maternal PUFA intake on milk content.^18^

In patients with ASDs, the administration of n-3 PUFAs may be of special importance, due to their role in brain structure and functionality, neurotransmission and biomembranes, among others.^20^ Even though limited benefits of n-3 PUFAs are reported for this population in randomized controlled trials, the absence of statistical significance is attributed to the small number of treated patients and the lack of clinical studies.^20^ However, PUFA supplementation may be suggested as a complement for other therapeutic approaches.^20^

On the other hand, the association between manifestations of ADHD and low circulating levels of n-3 PUFAs has been consistently reported in different observational studies summarized in a systematic review.^20^ Alterations in the serum profile of n-3 PUFAs are recognized in children and adults with ADHD, although it has not yet been defined whether these changes are a consequence of an inadequate intake or abnormalities in the metabolism of these fatty acids. In a meta-analysis,^20^ a favorable and significant effect of these supplements has been identified; as a consequence, their co-administration with the available pharmacological strategies may be suggested.

## Safety

The supplementation with N-3 PUFAs is considered a highly safe intervention, even regarding the maximum daily doses of 3 g to 4.5 g, according to regulatory agencies.^21^ Between 1% and 10% of the treated patients may report mild adverse events (dyspepsia, nausea, diarrhea, tendency to develop bleeding), while digestive adverse reactions only involve 0.1% to 1% of the treated patients. The gastrointestinal effects are usually improved with the administration with meals or with a gradual increase in dose.^21^ Even though isolated cases of increased transaminase levels have been reported, n-3 PUFAs appear to be associated with hepatoprotective effects, and their indication for the treatment of non-alcoholic liver steatosis has even been suggested.^21^

## Conclusion

The supplementation with n-3 PUFAs before, during and after pregnancy is linked to numerous maternal, fetal and childhood benefits regarding the reproductive capacity of women and the cognitive functionality of their offspring. The diffusion of the use of these supplements could improve the prognosis of these patients in a simple, effective way, and with high safety rates.
